# Supplementary material for: Heat adaptation of phage T7 under an extended genetic code
Source: Virus Evol. 2021 Dec 1;7(2):veab100. doi: 10.1093/ve/veab100 (PMC8923235; doi:10.1093/ve/veab100)
Supplement: veab100_Supp [file veab100_supp.zip › Sequencing_Analysis.zip!/Sequencing_Analysis/A2/evidence/JC_SIDE_2_51.html]

BRESEQ :: Evidence


| New junction evidence | | | | | | | | | | | |
| --- | --- | --- | --- | --- | --- | --- | --- | --- | --- | --- | --- |
|  | | seq id | position | reads (cov) | reads (cov) | score | skew | freq | annotation | gene | product |
| \* | ? | NC\_001604.1 | = 13455 | 2 (0.000) | 269 (0.600) | 158/586 | 0.3 | 94.6% | intergenic (–/–) | *–/–* | –/– |
|  | ? | NC\_001604.1 | 14331 = | 29 (0.070) |  |  |  |  | intergenic (–/–) | *–/–* | –/– |

Only 100 of 520 total aligned reads displayed.  

`AGAACTTAGGCTGGACTTGGACAAGACAGACTGGCTGGAAGAAACCAGAAGTTACCAAGCTGAGTCATAAGGTGTTCGATAAGGACACTATGACCCACATCAAGGCTGGTGATTGGGTTAAGGTTGACATGGGAGTTGTTGGTGGATACGGCTACGTCCGCTCAGTTAGTGGCAAATATGCACAAGTGTCATACATCACAGGTGTTACTCCACGCGGTGCAATCGTTGCCGATAAGACCAACATGATTCACACAGGTTTCTTGACAGTTGTTTCATATGAAGAGATTGTTAAGTCACGATAATCAATAGGAGAAATCAATATGATCGTTTCTGACATCGAAGCTAACGCCCTCTTAGAGAGCGTCACTAAGTTCCACTGCGGGGTTATCTACGACTACTCCACCGCTGAGTACGTAAGCTACCGTCCGAGTGACTTCGGTGCGTATCTGGATGCGCTGGAAGCCGAGGTTGCACGAGGCGGTCTTATTGTGTTCCACAACGGTCACAAGTATGACGTTCCTGCATTGACCAAACTGGCAAAGTTGCAATTGAACCGAGAGTTCCACCTTCCTCGTGAGAACTGTATTGACACCCTTG  >  NC_001604.1/14033‑14629`  

                                                                                                                                                                                                                                                                                                          `|`                                                                                                                                                                                                                                                                                                            
`acttacctacgtggttctggcgcactacgccaactatctgatactattattgcccttgagcgtaatcagcaaggcgatatgcctaaccttgtcctcgttcgtattctcaagtgccgctttactggtgatactggtatcgctggctacatggaatacaacaaggaaaccggatggcttgaaccatcaagttactcaggggaagaagagtcacactcagagtcaacagactggtccaacgacactgacttatgacaggattcttgaCAGTTGTTTCATATGAAGAGATTGTTAAGTCACGATa                                                                                                                                                                                                                                                                                                          <  1:46388‑M2/37‑1 (MQ=255)`  
      `gaatacaacaaggaaaccggatggcttgaaccatcaagttactcaggggaagaagagtcacactcagagtcaacagactggtccaatgacactgacttctgacaggattattgatgactttccagacgactacgagaagtttcgctggagagtcccattctaatacgactcactaaaggagacacaccatgttcaaactgattaagaagttaggccaactgctggttcgtatgtacaacgtggaagccaagcgactgaacgatgaggctcgtaaagaggccacacagtcacgATAATcaaa                                                                                                                                                                                                                                                                                                    >  1:724975‑M2/293‑300 (MQ=255)`  
         `cgtggttctggtgcactacgccaactatctgatactattattgccctttagcgtaatcagcaaggcgatatgcctaaccttgtcctcgttcgtattctcaagtgccgctttactggtgatactggtatcgctggctacatggaatacaacaaggaaaccggatggcttgaactatcaagttactcaggggaagaagagtcacactcagagtcaacagactggtccaacgacactgacttctgacaggattcttgaCAGTTGTTTCATATGAAGAGATTGTTAAGTCACGATAATCAATAgg                                                                                                                                                                                                                                                                                                 <  1:259009‑M2/46‑1 (MQ=255)`  
         `agatgtgtataagatacagggcttgaaccatcaagttactcaggggaagaagagtcacactcagagtcaacagactggtccaacgacactgacttctgacaggattcttgatgactttccagacgactacgagaagtttcgctgtagagtcccattctaatacgactcactaaaggagacacaccatgttcaaactgattaagaagttaggccaactgctggttcgtatgtacaacgtggaagccaagcgactgaacgatgaggctcgtaaagaggccacacagtcacgATAATCAATAgg                                                                                                                                                                                                                                                                                                 <  1:255591‑M2/12‑1 (MQ=255)`  
         `aagagacaggaaactggatggcttgaaccatcaagttactcaggggaagaagagtcacactcagagtcaacagactggtccaacgacactgacttctgacaggattcttgatgactttccagacgactacgagaagtttcgctggagagtcccattctaatatgactcactaaaggagacacaccatgttcaaactgattaagaagttaggccaactgcttgttcgtatgtacaacgtggaagccaagcgactgaacgatgaggctcgtaaagaggccacacagtcacgATAATCAATAgg                                                                                                                                                                                                                                                                                                 <  1:152775‑M2/12‑1 (MQ=255)`  
           `gtataagagacagctacgccaactatctgatactattattgcccttgagcgtaatcagcaaggcgatatgcctaaccttgtcctcgttcgtattctcaagtgccgcttgaatggtgatactggtatcgctggctacatggaatacaacaaggaaaccggatggcttgaaccatcaagttactcaggggaagaagagtcacactcagagtcaacagactggtccaacgacactgacttctgacaggtttcttgaCAGTTGTTTCATATGAAGAGATTGTTAAGTCACGATAATCAATAGgag                                                                                                                                                                                                                                                                                               <  1:670598‑M2/48‑1 (MQ=255)`  
           `ctcggagatgtgtataagagacagatctgatactattattgcccttgagcgtaatcagcaaggcgatatgcctaaccttgtcctcgttcgtattctcaagtgccgctttactggtgatactggtatcgctggctacatggaatacaacaaggaaaccggatggcttgaaccatcaagttactcaggggaagaagagtcacactcagagtcaacagactggtccaacgacactgacttctgacaggtttcttgaCAGTTGTTTCATATGAAGAGATTGTTAAGTCACGATAATCAATAGgag                                                                                                                                                                                                                                                                                               <  1:822261‑M2/48‑1 (MQ=255)`  
                     `accggatggcttgaaccatcaagttactcaggggaagaagagtcacactcagagtcaacagactggtccaacgacactgacttctgacaggattcttgatgactttccagacgactacgagaagtttcgctggagagtcccattctaatacgactaactaaaggagacacaccatgttcaaactgattaagaagttaggccaactgctggttcgtatgtacaacgtggaagccaagcgactgaacgatgaggctcgtaaagaggccacacagtcacgATAATCAATAGGAGAAATCAatat                                                                                                                                                                                                                                                                                     <  1:579385‑M2/24‑1 (MQ=255)`  
                        `ctacgccaactatctgatactattattgcccttgagcgtaatcagcaaggcgatatgcctaaccttgtcctcgttcgtattctcaagtgccgctttactggtgatactggtatcgctggctacatggaatacaacaaggaaaccggatggcttgaaccatcaagttactcaggggaagaagagtcacactcagagtcaacatactggtccaacgacactgacttctgacaggattcttgaCAGTTGTTTCATATGAAGAGATTGTTAAgtcacgataatcaatagctgtctcttatacaca                                                                                                                                                                                                                                                                                  >  1:230919‑M2/241‑285 (MQ=255)`  
                        `ctacgccaactatctgatactattattgcccttgagcgtaatcagcaaggcgatatgcctaaccttgtcctcgttcgtattctcaagtgccgctttactggtgatactggtatcgatggctacatggaatacaacaaggaaaccggatggcttgaaccatcaagttactcaggggaagaagagtcacactcagagtcaacagactggtccaacgacactgacttctgacaggattcttgaCAGTTGTTTCATATGAAGAGATTGTTAAGTCACGATAATCAATAGGAGAAatcaatactgt                                                                                                                                                                                                                                                                                  >  1:243850‑M2/241‑297 (MQ=255)`  
                        `ctacgccaactatctgatactattattgcccttgagcgtaatcagcaaggcaatatgcctaaccttgtcctcgttcgtattctcaagtgccgctttactggtgatactggtatcgctggctacatggaatacaacaaggaaaccggatggcttgaaccatcaagttactgaggggaagaagagtcacactcagagtcaacagactggtccaacgacactgacttctgacaggattcttgaCAGTTGTTTCATATGAAGAGATTGTTAAGTCACGATAATCAATAGGAGAAAtcaatatctg                                                                                                                                                                                                                                                                                  >  1:418477‑M2/241‑298 (MQ=255)`  
                        `acagctggcgcactacgccaactatctgatactattattgcccttgagcgtaatcagcaaggcgatatgcctaaccttgtcctcgttcgtattctcaagtgccgctttactggtgatactggtatcgctggctacatggaatacaacaaggaaaccggatggcttgaaccatcaagttactcaggggaagaagagtcactctcagagtcaacagactggtccaacgacactgacttctgaCAGTTGTTTCATATGAAGAGATTGTTAAGTCACGATAATCAATAGGAGAAATCAATAGGAt                                                                                                                                                                                                                                                                                  <  1:401140‑M2/61‑1 (MQ=255)`  
                            `ggcttgaaccatcaagttactcaggggaagaagagtcacactaagagtcaacagactggtccaacgacactgacttctgacaggattcttgatgactttccagacgactacgagaagtttcgctggagagtcccattctaatacgactcactaaaggagacacaccatgttcaaactgattaagaagttaggccaactgctggttcgtatgtacaacgtggaagccaagcgactgaacgatgaggctcgtaaagaggccacacagtcacgATAATCAATAGGAGAAATCAATATGATCGtt                                                                                                                                                                                                                                                                              >  1:466872‑M2/271‑301 (MQ=255)`  
                               `aactatctgatactattattgcccttgagcgtaatcagcaaggcgatatgcctaaccttgtcctcgttcgtattctcaagtgccgctttactggtgatactggtatcgctggctacatggaatacaacaaggaaaccggatggcttgaaccatcaagttactcaggggaagaagagtcacactcagagtcaacagactggtccaacgacactgacttctgacaggattcttgaCAGTTGTTTCATATGAAGAGATTGTTAAGTCacgataatcaataggagaaagcctttctcttatccac                                                                                                                                                                                                                                                                           >  1:750131‑M2/234‑284 (MQ=255)`  
                                  `tgggctcggagatgggtataagagacaggtcacactcatagtcaacagactggtccaacgacactgacttctgacaggattcttgatgactttccagacgactacgagaagtttcgctggagagtcccattctaatacgactcactaaaggagacacaccatgttcaaactgattaagaagttaggccaactgctggttcgtatgtacaacgtggaagccaatcgactgaacgatgaggctcgtaaagaggccacacagtcacgATAATCAATAGGAGAAATCAATATGATCATTTCTGAc                                                                                                                                                                                                                                                                        <  1:370033‑M2/37‑1 (MQ=255)`  
                                   `atctgatactattattgcccttgagcgtaatcagcaaggcgatatgcctaaccttgtcctcgttcgtattctcaagtgccgctttactggtgatactggtatcgctggctacatggaatacaacaaggaaaccggatggcttgaaccatcaagttactcaggggaagaagagtcacactcagagtcaacagactggtccaacgacacttacttctgacaggattcttgaCAGTTGTTTCATATGAAGAGATTGTTAAGTCACGATAATCAATAGTAGAAATCAATATGATCGTTTCTGACa                                                                                                                                                                                                                                                                       >  1:503375‑M2/230‑301 (MQ=255)`  
                                          `cgtgggctcggagatgtgtataagagacagggcgatatgcctaaccttgtcctcgttcgtattcacaagtgccgctttactggtgatactggtatcgctggctacatggaatacaacaaggaaaccggatggcttgaaccatcaagttactcaggggaagaagagtcacactcagagtcaacagactggtccaacgacactgacttctgacaggattcttgaCAGTTGTTTCATATGAAGAGATTGTTAAGTCACGATAATCAATATGAGAAATCAATATGATCGTTTCTGACATCAAAGc                                                                                                                                                                                                                                                                <  1:775838‑M2/79‑1 (MQ=255)`  
                                          `aatgtgtataagagacaggagtcacactcagagtcaacagactggtccaacgacactgacttctgacaggattcttgatgactttccagacgactacgagaagtttcgctggagagtcccattctaatacgactcactaaaggagacacaccatgttcaaactgattaagaagttaggccaactgctggttcgtatgtacaacgtggaagccaagcgactgaacgatgaggctcgtaaagaggccacacagtcacgATAATCAATAGGAGAAATCAATATGATCGTTTCTGACATCGAAGc                                                                                                                                                                                                                                                                <  1:64966‑M2/45‑1 (MQ=255)`  
                                             `attattgcccttgagcgtaatcagcaaggcgatatgcctaaccttgtcctcgttcgtattctcaagtgccgctttactggtgatactggtatcgctggctacatggaatacaacaaggaaaccggatggcttgaaccatcaagttactcagtggaagaagagtcacactcagagtcaacagactggtccaacgacactgacttcttacaggattcttgaCAGTTGTTTCATATGAAGAGATTGTTAAGTCACGATAATCAATAGGAGAAATCAATATGATCGTTTCTGACATCGAAGCTaa                                                                                                                                                                                                                                                             >  1:372987‑M2/220‑301 (MQ=255)`  
                                                 `caggggaagaagagtcacactcagagtcaacagactggtccaacgacactgacttctgacaggattcttgatgactttccagaagactacgagaagtttcgctggagagtcccattctaatacgactcactaaaggagacacaccatgttcaaactgattaagaagttaggccaactgctggttagtatgtacaacgtggaagccaagcgactgaacgatgaggctcgtaaagaggccacacagtcacgATAATCAATAGGAGAAATCAATATGATCGTTTCTGACATCGAAGCTAACGcc                                                                                                                                                                                                                                                         <  1:736649‑M2/52‑1 (MQ=255)`  
                                                  `cgtgggctcggagatgtgtataagagacaggcctaaccttgtcctcgttcgtattctcaagtgccgctttactggtgatactggtatcgctggctacatggaatacaacaaggaaaccggatggcttgaaccatcaagttactcaggggaagaagagtcacactcagagtcaacagactggtccaacgacactgacttctgacaggattcttgaCAGTTGTTTCATATGAAGAGATTGTTAAGTCACGATAATCAATAGGAGAAATCAATATGATCGTTTCTGACATCGAAGCTAACGccc                                                                                                                                                                                                                                                        <  1:414722‑M2/87‑1 (MQ=255)`  
                                                   `ggggaagaagagtcacactcagagtcaacagactggtccaacgacactgacttctgacaggattcttgatgactttccagacgactacgagaagtttcgctggcgagtcccattctaatacgactcactaaaggagacacaccatgttcaaactgattaagaagttaggccaactgctggttcgtatgtacaacgtggaagacaagcgactgaacgatgaggctcgtaaagaggccacacagtaacgATAATCAATCGGAGAACTCAATATGATCGTTTCTGCCATCGAAGCTAACGCCct                                                                                                                                                                                                                                                       >  1:84562‑M2/248‑301 (MQ=255)`  
                                                      `gaagaagagtcacactcagagtcaacagactggtccaacgacactgacttctgacaggattcttgatgactttccagacgactacgagaagtttcgctggagagtcccattctaatacgactcactaaaggagacacaccatgttcaaactgattaagaagttaggccaactgctggttcgtatgtacaacgtggaagccaagcgactgaacgatgaggctcgtaaagaggccacacagtcacgATAATCAATAGGAGAAATCAATATGATCGTTTCTGACATCGAAGCTAACGCCCTCtt                                                                                                                                                                                                                                                    <  1:765677‑M2/57‑1 (MQ=255)`  
                                                      `cttgagcgtaatcagcaaggcgatatgcctaaccttgtcctcgttcgtattctcaagtgccgctttactagtgatactggtatcgctggctacatggaatacaacaaggaaaccggatggcttgaaccatcaagttactcaggggaagaagagtcacactaagagtcaacagactggtccaacgacactgacttctgacaggattcttgaCAGTTGTTTCATATGAAGAGATTGTTAAGTCAAGATAATCAATAGGAGAAATCAATATGATCGTTTCTGACATCGAAGCTAACGCCCTCtt                                                                                                                                                                                                                                                    <  1:573087‑M2/91‑1 (MQ=255)`  
                                                       `ctattattgccctttagcgtaatcagcaaggcgatatgcctaaccttgtcctcgttcgtattctcaagtgccgctttactggtgatactggtatcgctggctacatggaatacaacaaggaaaccggatggcttgaaccatcaagttactcaggggaagaagagtcacactcagagtcaacagactggcccaacgacactgacttctgaCAGTTGTTTCATATGAAGAGATTGTTAAGTCACGATAATCAATAGGAGAAATCAATATGATCGTTTCTGACATCGAAGCTAACGCCCTCTTa                                                                                                                                                                                                                                                   >  1:334638‑M2/210‑301 (MQ=255)`  
                                                          `ttattgcccttgagcgtaatcagcaaggcgatatgcctaaccttgtcctcgttcgtattctcaagtgccgctttactggtgatactggtatcgctggctacatggaatacaacaaggaaaccggatggcttgaaccatcaagttactaaggggaagaagagtcacactcagagtcaacagactggtccaacgacactgacttctgaCAGTTGTTTCATATGAAGAGATTGTTAAGTCACGATAATCAATAGGAGAAATCAATATGATCGTTTCTGACATCGAAGCTAACGCCCTCTTagag                                                                                                                                                                                                                                                <  1:759505‑M2/95‑1 (MQ=255)`  
                                                          `gggagatgtgtataagatacaggcaaggcgatatgcctaaccttgtcctcgttcgtattctcaagtgccgctttactggtgatactggtatcgctggctacatggaatacaacaaggaaaccggatggcttgaaccatcaagttactcaggggaagaagagtcacactaagagtcaacagactggtccaacgacactgacttctgaCAGTTGTTTCATATGAAGAGATTGTTAAGTCACGATAATCAATAGGAGAAATCAATATGATCGTTTCTGACATCGAAGCTAACGCCCTCTTagag                                                                                                                                                                                                                                                <  1:306952‑M2/95‑1 (MQ=255)`  
                                                          `agcgtaatcagcaaggcgatatgcctaaccttgtcctcgttcgtattctcaagtgccgctttactggtgatactggtatcgctggctacatggaatacaacaaggaaactggatggcttgaaccatcaagttactcaggggaagaagagtcacactcagagtcaacagactggtccaacgacactgacttctgacaggattcttgaCAGTTGTTTCATATGAAGAGATTGATAAGTCACGATAATCAATAGGAGAAATCAATATGATCGTTTCTGACATCGAAGCTAACGCCCTCTTagag                                                                                                                                                                                                                                                <  1:816039‑M2/95‑1 (MQ=255)`  
                                                          `aatagtcaaactcagagtcaacagattggtccatcgacactgacttctgacaggtttcgtgatgactgtccagacgactacgagaagtgtcgctggagagtcccattctaatacgactcactaaaggagacacaccatgttcaaactgattaagaagttaggccaactgctggttcgtatgtacaacgtggaagccaagtgactgaacgatgaggctcgtaaagaggccacacagtcacgATAATCAATAGGAGAAATCAATATGATCGTTTCTGACATCGAAGCTAACGCCCTCTTagag                                                                                                                                                                                                                                                <  1:148403‑M2/61‑1 (MQ=255)`  
                                                          `aagagtcacactcagagtcaacagactggtccaacgacactgacttctgacaggattcttgatgactttccagacgactacgagaagtttcgctggatagtcccattctaatactactcactaaaggagacacaccatgttcaaactgattaagaagttaggccaactgctggttcgtatgtacaacgtggaagccaagcgactgaacgatgaggctcgtaaagaggccacacagtcacgATAATCAATAGGAGAAATCAATATGATCGTTTCTGACATCGAAGCTAACGCCCTCTTagag                                                                                                                                                                                                                                                <  1:236899‑M2/61‑1 (MQ=255)`  
                                                          `aagagtcacacgaagagtcaacagactggtccaacgacactgacttctgacaggattcttgatgactttccagactactacgagaagtttcgctggagagtcccattctaatacgactcactaaaggagacacaccatgttcaaactgattaagaagttaagccaactgctggttcgtatgtacaacgtggaagccaagcgactgaacgatgaggctcgtaaagaggccacacagtcacgATAATCAATAGTAGAAATCAATATGATCGTTTCTGACATCGAAGCTAACGCCCTCTTagag                                                                                                                                                                                                                                                <  1:13775‑M2/61‑1 (MQ=255)`  
                                                            `gctcggagatgtgtataatgtacagggtccaatgacactgacttctgacaggattcttgatgactttccagacgactacgagaagtttcgctggagagtcccattctaatacgactcactaaaggagacacaccatgttcaaactgattaagaagttaggccaactgctggttcgtatgtacaacgtggaagccaagcgactgaacgatgaggctcgtaaagaggccacacagtcacaATAATCAATAGGAGAAATCAATATGATCGTTTCTGACATCGAAGCTAACGCCCTCTTagagag                                                                                                                                                                                                                                              <  1:582456‑M2/63‑1 (MQ=255)`  
                                                            `cgtaagcagcaaggcgatatgcctaaccttgtcctcgttcgtattctcaagtgccgctttactggtgatactggtatcgctggctacatggaatacaacaaggaaaccggatggcttgaaccatcaagttactcaggggaagaagagtcacactcagagtcaacagactggtccaacgacactgacttctgacaggattcttgaCAGTTGTTTCATATGAAGAGATTGTTAAGTCACGATAATCAATAGGAGAAATCAATATGATCGTTTCTGACATCGAAGCTAACGCCCTCTTagagag                                                                                                                                                                                                                                              <  1:629231‑M2/97‑1 (MQ=255)`  
                                                                    `ctcagagtcaacagactggtccaacgacactgacttatgacaggattcttgatgactttccagacgactacgagaagtttcgatggagagtcccattctaatacgactcactaaaggagacacaccatgttcaaactgattaagaagttaggccaactgctggttcgtatgtacaacgtggaagccaagcgactgaacgatgaggctcgtaaagaggccacacagtcacgATAATCAATAGGAGAAATCAATATGATCGTTTCTGACATCGAAGCTAACGCCCTCTTAGAGAGCGTCACTa                                                                                                                                                                                                                                      <  1:285583‑M2/71‑1 (MQ=255)`  
                                                                          `tataagagacagccttgtcctcgttcgtattctcaagtgccgctttactggtgatactggtatcgcttgctacatggaatacaacaaggaaaccggatggcttgaaccatcaagttactcaggggaagaagagtcacactcagagtcaacagactggtccaacgacactgacttctgacaggtttcttgaCAGTTGTTTCATATGAAGAGATTGTTAAGTCACGATAATCAATAGGAGAAATCAATATGATCGTTTCTGACATCGAAGCTAACGCCCTCTTAGAGAGCGTCACTAAGTTcc                                                                                                                                                                                                                                <  1:535710‑M2/111‑1 (MQ=255)`  
                                                                            `cagtgcctaaccttgtcctcgttcgtattctcaagtgccgctttacttgtgatactggtatcgctggctacatggaatacaacaaggaaaccggatggcttgaaccatcaagttactcatgggaagaagagtcacactcagagtcaacagactggtccaacgacactgacttctgacaggattcttgaCAGTTGTTTCATATGAAGAGATTGTTAAGTCACGATAATCAATAGGAGAAATCAATATGATCGTTTCTGACATCGAAGCTAACGCCCTCTTAGAGAGCGTCACTAAGTTCCAc                                                                                                                                                                                                                              >  1:542253‑M2/189‑301 (MQ=255)`  
                                                                            `caacagactggtccaacgacactgacttctgacaggattcttgatgactttccagacgactacgagaagtttcgctggagagtcccattctaatacgactcactaaaggagacacaccatgttcaaactgattaagaagttaggccaactgctggttcgtatgtacaacgtggaagccaagcgactgaacgatgaggctcgtaaagaggccacacagtcacgATAATCAATAGGAGAAATCAATATGATCGTTTCTGACATCGAAGCTAACGCCCTCTTAGAGAGCGTCACTAAGTTCCAc                                                                                                                                                                                                                              <  1:106750‑M2/79‑1 (MQ=255)`  
                                                                            `caacagactggtccaacgacactgacttctgacaggattcttgatgactttccagacgactacgagaagtttcgctggagagtcccattctaatacgactcactaaaggagacacaccatgttcaaactgattaagaagttaggcaaactgctggttcgtatgtacaacgtggaagccaagcgactgagcgatgaggctcgtaaagaggccacacagtcacgATAATCAATAGGAGAAATCAATATGATCGTTTCTGACATcgaagctaacgccctcttagagctgtctcttatacacatt                                                                                                                                                                                                                              >  1:476419‑M2/223‑283 (MQ=255)`  
                                                                            `aagagacagaccttgtcctcgttcgtattctcaagttccgctttactggtgatactggtatcgctggctacatggaatacaacaaggaaaccggatggcttgaaccatcaagttactcaggggaagaagagtcacactcagagtcaacagactggtccaacgacactgacttctgacaggattcttgaCAGTTGTTTCATATGAAGAGATTGTTAAGTCACGATAATCAATAGGAGAAATCAATATGATCGTTTCTGACATCGAAGCTAACGCCCTCTTAGAGAGCGTCACTAAGTTCCAc                                                                                                                                                                                                                              <  1:208135‑M2/113‑1 (MQ=255)`  
                                                                              `acagactggtccaacgacactgacttcggacaggattcttgatgactttccagacgactacgagaagtttcgctggagagtcccattctaatacgactcactaaaggagacacaccatgttcaaactgattaagaagttaggccaactgctggttcgtatgtacaacgtggaagccaagcgactgaacgatgaggctcgtaaagaggccacacagtcacgATAATCAATAGGAGAAATCAATATGATCGTTTCTGACATCGAAGCTAACGCCCTCTTAGAGAGCGTCACTAAGTTCCACTg                                                                                                                                                                                                                            <  1:589186‑M2/81‑1 (MQ=255)`  
                                                                                `gcctaaccttgtcctcgttcgtattctcaagtgccgctttactggtgatactggtatcgatggctacatggaatacaacaaggaaaccggatggcttgaaccatcaagttactcaggggaagaagagtcacactcagagtcaacagactggtccaacgacactgacttctgacaggattcttgaCAGTTGTTTCATATGAAGAGATTGTTAAGTCACGATAATCAATAGGAGAAATCAATATGATCGTTTCTGacatcgaagctaacgccctcttagagctgtctcttatacacatctccg                                                                                                                                                                                                                          >  1:455247‑M2/185‑279 (MQ=255)`  
                                                                                 `gactcgtccaacgacactgacttctgacaggattcttgattactttccagacgactacgagaagtttcgctggagagtcccattctaatacgactcactaaatgagacacaccatgttcaaactgattaagaagttaggccaactgctggttcgtatgtacaacgtggaagccaagcgactgaacgatgaggctcgtaaagaggccacacagtcacgATAATCAATAGGAGAAATCAATATGATCGTTTCTGACATCGAAGCTAACGCCCTCTTAGAGAGCGTCACTAAGTTCCACtgctg                                                                                                                                                                                                                         >  1:44624‑M2/218‑299 (MQ=255)`  
                                                                                    `tggtccaacgacactgacttctgacaggattcttgatgactttccagacgactacgagaagtatcgctggagagtcccattctaatacgactcactaaaggagacacaccatgttcaaactgattaggaagttaggccaactgctggttcgtatgtacaacgtggaagccaagcgactgaacgatgaggctcgtaaagaggccacacagtcacgATAATCAATAGGAGAAATCAATATGATCGTTTCTGACATCGAAGCTAACGCCCTCTTAGAGAGCGTCACTAAGTTCCACTGCGGGGt                                                                                                                                                                                                                      <  1:599708‑M2/87‑1 (MQ=255)`  
                                                                                            `gcctaaccttgtcctcgttcgtattctcaagtgccgctttactggtgatactggtatcgctggctacatggaatacaacaaggaaaccggatggcttgaaccatcaagttactcagggtaagaagagtcacactcagagtcaacagactggtccaacgacactgacttctgaCAGTTGTTTCATATGAAGAGATTGTTAAGTCACGATAATCAATAGGAGAAATCAATATGATCGTTTCTGACATCGAAGCTAACGCCCTCTTAGAGAgcgtcactaagttcaacctgtctcttatacaca                                                                                                                                                                                                              >  1:253344‑M2/173‑285 (MQ=255)`  
                                                                                                   `ggctcggagatgtgtataagagacagtgatactggtatcgctggctacatggaatacaacaaggaaaccggatggcttgaaccatcaagttactcaggggaagaagagtcacactcagagtcaacagactggtccaacgacactgacttctgacaggattcttgaCAGTTGTTTCATATGAAGAGATTGTTAAGTCACGATAATCAATAGGAGAAATCAATATGATCGTTTCTGACATCGAAGCTAACGCCCTCTTAGAGAGCGTCACTAAGTTCCACTGCGGGGTTATCTACGACTACTc                                                                                                                                                                                                       <  1:101125‑M2/136‑1 (MQ=255)`  
                                                                                                    `gacttctgacaggattcttgatgactttccagacgactacgagaagtttcgctggagagtcccattctaatacgactcactaaaggagacacaccatgttcaaactgattaagaagtaggccaactgctggttcgtatgtacaacgtggaagccaagcgactgaacgatgaggctcgtaaagaggccacaaagtcacgATAATCAATAGGAGAAATCAATATGATCATTTCTGACCTCGAAGCTAACGCCCTCTTAAAGAGCGTCCCTAAGTTCCACTGCGGGATTATCTCAGACTACTcc                                                                                                                                                                                                      >  1:696410‑M2/199‑301 (MQ=255)`  
                                                                                                        `ctgacaggattcttgatgactttccagacgactacgagaagtttcgctggagagtcccattctaatacgactcactaaaggagacacaccatgttcaaactgattaagaagttaggccaactgctggttcgtatgtacaacgtggaagccaagcgactgaacgatgaggctcgtaaagaggccacacagtaacgATAATCAATAGGAGAAATCAATATGATCGTTTCTGACATCGAAGCTAACGCCCGCTTAGAGAGCGTAACTAAGTTCCACTGCGGGGTTATCTAAGACTACTCTaccc                                                                                                                                                                                                  >  1:782031‑M2/195‑300 (MQ=255)`  
                                                                                                         `ctcaagtgccgctttactggtgatactggtatcgctggctacatggaatacaacaaggaaaccggatggcttgaaccatcaagttactcagggtaagaagagtcacactcagagtcaacagactggtccaacgacactgacttctgacaggattctttaCAGTTGTTTCATATGAATAGATTGTTAAGTCACGATAATCAATATGAGAAATCAATATGATCGTTTCTGACATCGAAGCTAACGCCCTCTTAGAGAGCGTCACTAAGTTCCACTGCTGGGTTATCTACGACTACTCCACCGc                                                                                                                                                                                                 <  1:738433‑M2/142‑1 (MQ=255)`  
                                                                                                                 `ttcttgatgactttccagactactacgagaagtttcgctggagagtcccattctaatacgactcactaaaggagacacaccatgttcaaactgattaagaagttaggccaactgctggttcgtatgtacaacgtggaagccaagcgactgaacgatgaggctcgtaaagaggccacacagtcacgATAATCAATAGGAGAAATCAATATGATCGTTTCTGACATCGAAGCTAACGCCCTCTTAGAGAGCGTCACTAAGTTACACTGCGGGGTTATCTACGACTACTCCACCGCTGAGTACg                                                                                                                                                                                         <  1:117137‑M2/116‑1 (MQ=255)`  
                                                                                                                 `ttcttgatgactttccagacgactacgagaagtttcgctggagagtcccattctaatacgactcactaaaggagacacaccatgttcaaactgattaagaagttaggccaactgctggttcgtatgtacaacgtggaagccaagcgactgaacgatgaggctcgtaaagaggccacacagtcacgATAATCAATAGGAGAAATCAATATGATCGTTTCTGACATCGAAGCTAACGCCCTCTTAGAGAGCGTCACTAAGttccactgcggggttatctacgctgtctcttatacacatctcc                                                                                                                                                                                         >  1:637741‑M2/186‑280 (MQ=255)`  
                                                                                                                     `tcggagatgtgtataagagaaagtactggtatcgctggctacatggaatacaacaaggaaaccggatggcttgaaccatcaagttactcaggggaagaagagtcacactcagagtcaacagactggtccaacgacactgacttctgaCAGTTGTTTCATATGAAGAGATTGTTAAGTCACGATAATCAATAGGAGAAATCAATATGATCGTTTCTGACATCGAAGCTAACGCCCTCTTAGAGAGCGTCACTAAGTTCCACTGCGGGGTTATCTACGACTACTCCACCGCTGAGTACGTAAg                                                                                                                                                                                     <  1:677385‑M2/154‑1 (MQ=255)`  
                                                                                                                           `ggtgatactggtatcgctggctacatggaatacaacaaggaaaccggatggcttgaaccatgaagttactcaggggaagaagagtcacactcagagtcaacagactggtccaacgacactgacttctgacaggattcttgaCAGTTGTTTCATATGAAGAGATTGTTAAGTCACGATAATCAATAGGAGAAATCAATATGATCGTTTCTGACATCGAAGCTAACGCCCTCTTAGAGAGCGTCACTAAGTTCCACTGCGGGGTTAtctacgactactcccccgcctttctcttctacacatc                                                                                                                                                                               >  1:427206‑M2/142‑283 (MQ=255)`  
                                                                                                                               `atactggtatcgctggctacatggaatacaacaaggaaaccggatggcttgaaccatcaagttactcaggggaagaagagtcacactcagagtcaacagactggtccaacgacactgacttctgacagtattcttgaCAGTTGTTTCATATGAATAGATTGTTAAGTCACGATAATCAATAGGAGAAATCAATATGATCGTTTCTGACATCGAAGCTAACGCCCTCTTAGAGAGCGTCACTAAGTTCCACTGCGGGGTTATCTACGACTACTCCACCGCTGAGTACGTAAGCTACCgtctg                                                                                                                                                                           >  1:289552‑M2/138‑299 (MQ=255)`  
                                                                                                                                 `gacagactacgagaagtttcgctggagagtcccattctaatacgactcactaaaggagacacacaatgttcaaactgattaagaagttaggccaactgctggttcgtatgtacaacgtggaagccaagcgactgaacgatgaggctcgtaaagaggccacacagtcacgATAATCAATAGGAGAAATCAATATGATCGTTTCTGACATCGAAGCTAACGCCCTCTTAGAGAGCGTCACTAAGTTCCACTGCGGGGTTATCTACGACTACTCCACCGATGAGTACGTAAGCTACCGTCCGAg                                                                                                                                                                         <  1:480834‑M2/132‑1 (MQ=255)`  
                                                                                                                                 `agacgactacgagaagtttcgctggagagtcccattctaatacgactcactaaaggagacacaccatgttcaaactgattaagaagttaggccaactgctggttcgtatgtacaacgtggaagccaagcgactgaacgatgaggctcgtcaagaggccacacagtcacgATAATCAATAGGAGAAATCACTATGATCGTTTCTGACATCGAAGCTAACGCCCTCTTAGAGAGCGTCACTAAGTTCCCCTGCGGGGTTATCTACGACTACTCCCCCGCTGAGTACGtaagctacctgtctct                                                                                                                                                                         >  1:382399‑M2/170‑294 (MQ=255)`  
                                                                                                                                     `gtagcgctggctacatggaatacaacaaggaaaccggagggcttgaaccatcaagttactcaggggaagaagagtcacactcagagtcaacagactggtccaacgacactgacttctgacaggattcttgaCAGTTGTTTCATATGAAGAGATTGTTAAGTCACGATAATCAATAGGAGAAATCAATATGATCGTTTCTGACATCGAAGCTAACGCCCTCTTATAGAGCGTCACTAAGTTCCACTGCGGGGTTATCTACGACTACTCCACCGCTGAGTACGTAAGCTACCGTCCGAGTGAc                                                                                                                                                                     <  1:396011‑M2/170‑1 (MQ=255)`  
                                                                                                                                          `gctggctacatggaatacaacaaggaaaccggatggcttgaaccatcaagttactcaggggaagaagagtcacactcagagtcaacagactggtccaacgacactgacttctgacaggattcttgaCAGTTGTTTCATATGAAGAGATTGTTAAGTCACGATAATCAATAGGAGAAATCAATATGATCGTTTCTGACATCGAAGCTAACGCCCTCTTAGAGAGCGTCACTAAGTTCCACTGCGGGGTTATCTACGACTACTCCACCGCTGAGTACGTAAGCTACCGTCAGAGTGACTTCgg                                                                                                                                                                <  1:111998‑M2/175‑1 (MQ=255)`  
                                                                                                                                            `agaagtttcgctggagagtcccattctaatacgactcacgaaaggagacacaccatggtcaaactgattaagaagttaggccaactgctggttcgtatgtaaaacgtggaagccaagcgactgaacgatgaggctcgtaaagaggccacacagtcacgATAATCAATAGGAGAAATCAAAATGATCGTTTCTGACATCGAAGCTAACGCCCTCTTAGAGAGCGTCACTAAGTTCCACTGCGGGGTTATCTACGACTACTCCACCGCTGAGTACGTAAGCTACCGTCCGAGTGACTTCGGTg                                                                                                                                                              <  1:616259‑M2/143‑1 (MQ=255)`  
                                                                                                                                                  `tgcgctggagagtcccattctaatacgactcactaaaggatacacaccatgttcaaactgattaagaagttaggccaactgctggttcgtatgtacaacgtggaagccaagcgactgaacgatgaggctcgtaaagaggccacacagtcacgATAATCAATAGGAGAAATCAATATGATCGTTTCTGACATCGAAGCTAACGCCCTCTTAGAGAGCGTCACTAAGTTCCACTGCGGGGTTATCTACGACTACTCCACCGCTGAGTACGTAAGCTACCGTCCGAGTGACTTCGGTGCGTATc                                                                                                                                                        <  1:656512‑M2/149‑1 (MQ=255)`  
                                                                                                                                                  `ggctcggagatgtgtataagagacagggcttgaaccatcaagttactcaggggaagaagagtcacactcagagtcaacagactggtccaacgacactgacttctgacaggattcttgaCAGTTGTTTCATATGAAGAGATTGTTAAGTCACGATAATCAATAGGAGAAATCAATATGATCGTTTCTGACATCGAAGCTAACGCCCTCTTAGAGAGCGTCACTAAGTTACACTGCGGGGTTATCTACGACTACTCCACCGCTGAGTACGTAAGCTACCGTCCGAGTGACTTCGGTGCATATc                                                                                                                                                        <  1:745950‑M2/183‑1 (MQ=255)`  
                                                                                                                                                      `gctggctaaatggaatacaaaaaggaaaccggatggcttgaaccatcaagttactcaggggaagaagagtcacactcagagtcaacagactggtccaacgacactgacttctgaCAGTTGTTTCATATGAAGAGATTGTTAAGTCACGATAATCAATAGGAGAAATCAATATGATCGTTTCTGACATCGAAGCTAACGCCCTCTTAGAGAGCGTCACTAAGTTCCACTGCGGGGTTATCTACGACTACTCCACCGCTGAGTACGTAAGCTACCGTCCGAGTGACTTCGGTGCGTATCTGGa                                                                                                                                                    <  1:182938‑M2/187‑1 (MQ=255)`  
                                                                                                                                                      `ctggagagtcccattctaatacgactcactaaaggagacacaccatgttcaaactgattaagaagttaggccaactgctggttcgtatgtacaacgtggaagccaagctactgaacgatgaggctcgtaaagagtccacacagtcacgATAATCAATAGGAGAAATCAATATTATCGTTTCTGACATCGAAGCTAACGCCCTCTTAGAGAGCGTCACTAAGTTCCACTGCGTGGTTATCTACGACTACTCCACCGCTGAGTACGTAAGCTACCGTCCGAGTGACTTCGGTGCGTATCTGGa                                                                                                                                                    <  1:710026‑M2/153‑1 (MQ=255)`  
                                                                                                                                                        `ggagagtcccattctaatacgactcactaaaggagacacaccatgttcaaactgattaagaagttaggccaactgctggttcgtatgtacaacgtggaagccaagcgactgaacgataaggctcgtaaagaggccacacagtcacgATAATCAATAGGAGAAATCAATATGATCGTTTCTGACATCGAAGCTAAAGCCCTCTTAGAGAGCGTCACTAAGTTCCACTGCGGGGTTATCTACGACTACTCCACCGCTGAGTACGTAAGCTACCGTCCGAGtgacttcggtgctgtctcttata                                                                                                                                                  >  1:498714‑M2/147‑290 (MQ=255)`  
                                                                                                                                                            `aatccaattctaaaacgaatcaataaaggagacacaccatgttcaaactgattaagaagttaggccaactgctggttcgtatgtacaacgtggaagccaagcgactgaacgatgaggctcgtaaagaggccacacagtcaagATAATCAATAGGAGAAATCAATATGAGCGTTTCTGAAATCGAAGCTAACGCCCGCTTAGAGAGCGTCACTAAGTTCCACTGCGGGGTTATCTACGACTACTCCACCGCTGAGTACGTAAGCTACCGTCCGAGTGACTTCGGTGCGTATCTGGATGCGCt                                                                                                                                              <  1:353102‑M2/159‑1 (MQ=255)`  
                                                                                                                                                             `gtcccattctaatacgactcactaaaggagacacaccatgttcaaactgattaagaagttaggccaactgctggttcgtatgtacaacgtggaagccaagcgactgaacgatgaggctcgtaaagaggccacacagtcacgATAATCAATAGGAGAAATCAATATGATCGTTTCTGACATCGAAGCTACCGCCCTCTTCGAGAGCTTCACTAAGTTCCACTGCGTGGTTATCTACGACTACTCCACCGCTGCGTACGTAAGCTACCGTCCGAGTGACTTCGGTGCGTATCTGGatgcgcct                                                                                                                                             >  1:298454‑M2/142‑299 (MQ=255)`  
                                                                                                                                                                   `ttctaatacgactcactaaaggagacacaccatgttcaaactgattaagaagttaggccaactgctggttcgtatgtacaacgtggaagccaagcgactgaacgatgaggctcgtaaagaggccacacagtaacgATAATCAATAGGAGAAATCAATATGATCGTTTCTGACATCGAAGCTAACGCCCTCTTAGAGAGCGTCACTAAGTTCCACTGCGGGGTTATCTACGACTACTCCACCGCTGAGTACGTAAGCTACCGTCCGAGTGACTTCGGTGCGTATCTGGATGCGCTGGAAGcc                                                                                                                                       >  1:552433‑M2/136‑301 (MQ=255)`  
                                                                                                                                                                     `ctaatacgactcactaaaggagacacaccatgttcaaactgattaagaagttaggccaactgctggttagtatgtacaacgtggaagccaagcgactgaacgatgaggctcgtaaagaggccacacagtcacgATAATCAATAGGAGAAATCAATATGATCGTTTCTGACATCGAAGCTAACGCCATCTTAGAGAGCGTCACTAAGTTCCACTGCGGGGTTATCTACGACTACTCCACCGCTGAGTACGTAAGCTACCGTCCGAGTGACTTCGGTGCGTATCTGGATGCGCTGGAAGCcgg                                                                                                                                     >  1:686288‑M2/134‑300 (MQ=255)`  
                                                                                                                                                                             `tcttgaaccatcaagttactcaggggaagaatagtcacactcagagtcaacagactggtccaacgacactgacttctgacagtattcttgaCAGTTGTTTCATATGAAGAGATTGTTAAGTCACGTTAATCAATAGGAGAAATCAATATGATCGTTTCTTACATCGAAGCTAACGCCCTCTTAGAGAGCGTCACTAAGTTCCACTGCGGGGTTATCTACGACTACTCCACCGCTGAGTACGTAAGCTACCGTCCGAGTGACTTCGGTGCGTATCTGGATGCGCTGGAAGCCGAGGTTGCAc                                                                                                                             >  1:178815‑M2/92‑301 (MQ=255)`  
                                                                                                                                                                             `actcactaaaggagacacaccatgttcaaactgattaataagttaggccaactgctggttcgtatgtacaacgtggaagccaagcgactgaacgatgaggctcgtaaagaggccacacagtcacgATAATCAATAGGATAAATCAATATGATCGTTTCTGACATCGAAGCTAACGCCCTCTTAGAGAGCGTCACTAAGTTCCACTGCGGGGTTATCTACGACTACTCCACCGCTGAGTACGTAAGCTACCGTCCGAGTGACTTCGGTGCGTATCTGGATGCGCTGGAAGCCGAGGTTGAAc                                                                                                                             >  1:19596‑M2/126‑301 (MQ=255)`  
                                                                                                                                                                                `cactaaaggagacacaccatgttcaaactgattaagaagttaggccaactgctggttcgtatgtacaacgtggaagccaagcgactgaacgatgaggctcgtaaagaggccacacagtcacgATAATCAATAGGAGACATCAATATGATCGTTTCTGACATCGAAGCTAACGCCCTCTTAGAGAGCTTCACTAAGTTCCACTGCGGGGTTATCTACGACTACTCCACCGCTGAGTACGTAAGCTACCGTCCGAGTgactttggtgcgtatctggatgcgcctgtctcttatacacatctcc                                                                                                                          >  1:329705‑M2/123‑280 (MQ=255)`  
                                                                                                                                                                                 `cctaaaggagacacaccatgttcaaactgattaagaagttaggccaactgctggttcgtatgtacaacgtggaagccaagcgactgaacgatgaggctcgtaaagaggccacacagtcacgATAATCAATAGGAGAAATCAATATGATCGTTTCTGACATCGAAGCTAACGCCCTCTTAGAGAGCGTCACTAAGTTCCACTGCGGGGTTATCTACGACTACTCCACCGCTGAGTACGTAAGCTACAGTCCGAGTGACTTCGGTGCGTATCTGGATGCGCTGGAAGCCGAGGTTGCACGAgg                                                                                                                         <  1:193059‑M2/180‑1 (MQ=255)`  
                                                                                                                                                                                          `gacacaccctgttcaaactgattaagaagttaggccaactgctggttcgtatgtacaacgtggaagccaagcgactgaacgatgaggctcgtaaagatgccacacagtcacgATAATCAATAGTAGAAATCAATATGCTCGTTTCTGACATCGAAGCTAACGCCCTCTTAGAGAGCGTCACTAAGTTCCACTGCGGGGTTATCTACGACTACTCCACCGCTGAGTACGTAAGCTACCGTCCGAGTGACTTCGGTGCGTATCTGGATGCGCTGGAAGCCGAGGTTGCACGAGGCGGTCTTAt                                                                                                                >  1:320253‑M2/113‑301 (MQ=255)`  
                                                                                                                                                                                            `ttactcaggggaagaagagtcacactcagagtcaacagactggtccaacgacactgacttctgacaggattcttgaCAGTTGTTTCATATGAAGAGATTGTTAAGTCACGATAATCAATAGGAGAAATCAATATGATCGTTTCTGACATCGAAGCTAACGCCCTCTTAGAGAGCGTCACTAAGTTCCACTGCGGGGTTATCTACGACTACTCCACCGCTGAGTACGTAAGCTACCGTCCGAGTGACTTCGGTGCGTATCTGGATGCGCTGGAAGCCGAGGTTGCACGAGGCGGTCTTATtg                                                                                                              <  1:158339‑M2/225‑1 (MQ=255)`  
                                                                                                                                                                                            `cacacccgtttaaaactgattaagaagttaggccaactgctggttcgtatgtacaacgtggaagccaagcgactgaacgatgaggcgcgtaaagaggccacacagtcacgATAATCAATAGGAGAAATCAATATGATCGTTTCTGACATCGAAGCTAACGCCCTCTGAGAGAGCGTCACTAAGTTCCACTGCGGGGTTATCTACGACTACTCCACCGCTGAGTACGTAAGCTACCGTCCGAGTGACTTCGGTGCGTATCTGGATGCGCTGGAAGCCGAGGTTGCACGAGGCGGTCTTATtg                                                                                                              <  1:386830‑M2/191‑1 (MQ=255)`  
                                                                                                                                                                                            `cacaccatgttcaaactgattaagaagttaggccaactgctggttcgtatgtacaacgtggaagccaatcgactgaacgatgaggctcgtaaagaggccacacagtcacgATAATCAATAGGAGAAATCAATATGATCGTTTCTGACATCGAAGCTAACGCCCTCTTAGAGAGCGTCACTAAGTTCCACTGCGGGGTTATCTACGACTACTCCACCGCTGAGTACGTAAGCTACCGTCCGAGTGACTTCGGTGCGTATCTGGATGCGCTGGAAGCCGAGGTTGCACGAGGCGGTCTTATtg                                                                                                              <  1:562514‑M2/191‑1 (MQ=255)`  
                                                                                                                                                                                            `cacaccatgttcaaactgattaagaagttaggccaactgctggttcgtatgtacaacgtggaagacaagcgactgaacgatgaggctcgtaaagaggccacacagtcacgATAATCAATAGGAGAAATCAATATGATCGTTTCTGACATCGAAGCTAACGCCCTCTTAGAGAGCGTCACTAAGTTCCACTGCGGGGTTATCTACGACTACTCCACCGCTGAGTACGTAAGCTACCGTCCGAGTGACTTCGGTGCGTATCTGGATGCGCTGGAAGCCGAGGTTGCACGAGGCGGTCTTATtg                                                                                                              <  1:728597‑M2/191‑1 (MQ=255)`  
                                                                                                                                                                                            `atgtgtataagagacaggattaagaagttaggccaactgctggttcgtatgtacaacgtggaagccaagcgactgaacgatgaggctcgtaaagaggccacacagtcacgATAATCAATAGGAGAAATCAATATGATCGTTTCTGACATCGAAGCTAACGCCCTCTTAGAGAGCGTCACTAAGTTCCACTGCGGGGTTATCTACGACTACTCCACCGCTGAGTACGTAAGCTACCGTCCGAGTGACCTCGGTGCGTATCTGGATGCGCTGGAAGCCGAGGTTGCACGAGGCGGTCTTATtg                                                                                                              <  1:93601‑M2/191‑1 (MQ=255)`  
                                                                                                                                                                                            `aagacagttttcaaactgattaagaagttaggccaacttctggttcgtatgtacaactttgaagccaagcgactgaacgatgaggctcgtaaagatgccacacagtcacgATAATCTATATGAGAAATCAATATGATCGTTTCTGACATCGAAGCTAACGCCCTCTTAGAGAGCGTCACTAAGTTCCACTGCGGGGTTATCTACGACTACTCCACCGCTGAGTACGTAAGCTACCGTCCGAGTGACTTCGGTGCGTATCTGGATGCGCTGGAAGCCGAGGTTGCATGAGGCGGTCTTATtg                                                                                                              <  1:43803‑M2/191‑1 (MQ=255)`  
                                                                                                                                                                                              `caccatgttcaaactgattaagaagttaggccaactgctggttcgtatgtacaacgtggaagccaagcgactgaacgatgaggctcgtaaagaggccacacagtcacgATAATCAATAGGAGAAATCAATATGATCGTTTCTGACATCGAAGCTAACGCCCTCTTAGAGAGCGTCACTAAGTTCCACTGCGGGGTTATCTACGACTACTCCACCGCTGAGTACGTAAGCTACCGTCCGAGTGACTTCGGTGCgtatctggatgcgctggaagccgagctgtctcttatacacatctccgag                                                                                                            >  1:229762‑M2/109‑277 (MQ=255)`  
                                                                                                                                                                                                 `caggggaagaagagtcacactcatagtcaacagactggtccaacgacactgacttctgacaggattcttgaCAGTTGTTTCATATGAAGAGATTGTTAAGTCACGATAATCAATAGGAGAAATCAATATGATCGTTTCTGACATCGAAGCTAACGCCCTCTTAGAGAGCGTCACTAAGTTCCACTGCGGGGTTATCTACGACTACTCCACCGCTGAGTACGTAAGCTACCGTCCGAGTGACTTCGGTGCGTATCTGGATGCGCTGGAAGCCGAGGTTGCACGATGCGGTCTTATTGTGTTc                                                                                                         <  1:704131‑M2/230‑1 (MQ=255)`  
                                                                                                                                                                                                    `gttcaaactgattaagaagttaggccaactgctggttcgtatgtaaaacgtggaagccaagcgactgaacgatgaggctcgtaaagaggccacacagtcacgATAATCAATAGGAGAAATCAATATGATCGTTTCTGACATCGAAGCTAACGCCCTCTTAGAGAGCGTCACTAAGTTCCACTGCGGGGTCATCTACGACTACTCCACCGCTGAGTACGTAAGCTACCGTCCGAGTGACTTCGGTGCGTATCTGGATGCGCTTGAAGCCGAGGTTGCACGAGGCGGTCTTATTGTGTGCcac                                                                                                      <  1:525263‑M2/199‑1 (MQ=255)`  
                                                                                                                                                                                                                          `gtcccactcagagtcaacagactggtccaacgacactgacttctgaCAGTTGTTTCATATGAATAGATTGTTAAGTCACGATAATCAATAGGAGAAATCAATATGATCGTTTCTGACATCGAAGCTAACGCACTCTTAGAGAGTGTCACTAAGTTCCACTGCGGGGTTATCTACGACTACTCCACCGCTGAGTACGTAAGCTACCGTCCGAGTGACTTCGGTGCGTATCTGGATGCGCTGGAAGCCGAGGTTGCACGAGGCGGTCTTATTGTGTTCCACAACGGTCACAAGTATGACGTTc                                                                                >  1:215346‑M2/47‑301 (MQ=255)`  
                                                                                                                                                                                                                               `ctcagagtcaacagactggtccaacgacactGATTTC‑TGACAGTTGTTTCATATGAAGAGATTGTTAAGTCACGATAATCAATAGGAGAAATCAATATGATCGTTTCTGACATCGAAGCTAACGCCCTCTTAGAGAGCATCACTAAGTTCCACTGCGGGGTTATCTACGACTACTCCACCGCTGAGTACGTAAGCTACCGTCCGAGTGACTTCGGTGCGTATCTGGATGCGCTGGAAGCCGAGGTTGCACGAGGCGGTCTTATTGTGTTCCACAACGGTCACAAGTATGACGTTCCTGCAt                                                                          <  1:88141/275‑1 (MQ=255)`  
                                                                                                                                                                                                                                `tgtgtataagagacagactgacttctgacaggattcttgaCAGTTGTTTCATATGAAGAGATTGTTAAGTCACGATAATCAATAGGAGAAATCAATATGATCGTTTCTGACATCGAAGCTAACGCCCTCTTAGAGAGCGTCACTAAGTTCCACTGCGGTGTTATCTACGACTACTCCACCGCTGAGTACGTAAGCTACCGTCCGAGTGACTTCGGTGCGTATCTGGATGCGCTGGAAGCCGAGGTTGCACGAGGCGGTCTTATTGTGTTCCACAACGGTCACAAGTATGACGTTCCTGCAt                                                                          <  1:609111‑M2/261‑1 (MQ=255)`  
                                                                                                                                                                                                                                `ctgctggttcgtatgtacaacgtggaagccaagcgactgaacgatgaggctcgtaaagaggccacacagtcacgATAATCAATAGGAGAAATCAATATGATCGTTTCTGACATCGAAGCTAACGCCCTCTTAGAGAGCGTCACTAAGTTCCACTGCGGGGTTATCTATGACTACTCCACCGCTGAGTACGTAAGCTACCGTCCGAGTGACTTCGGTGCGTATCTGGATGCGCTGGAAGCCGAGGTTGCACGAGGCGGTCTTATTGTGTTCCACAACGGTCACAAGTATGACGATCCTGCAt                                                                          >  1:790966‑M2/75‑301 (MQ=255)`  
                                                                                                                                                                                                                                `aagagacagcgtatgtacaacgtggaagccaagcgactgaacgatgaggctcgtaaagaggccacacagtcacgATAATCAATAGTAGAAATCAATATGATCGTTTCTGACATCGAAGCTAACGCCCTCTTAGAGAGCGTCACTAAGTTCCACTGCGGGGTTATCTACGACTACTCCACCGCTGAGTACGTAAGCTACCGTCCGAGTGACTTCGGTGCGTATCTGGATGCGCTGGAAGCCGAGGTTGCACTAGGCGGTCTTATTGTGTTCCACAACGGTCACAAGTATGACGTTCCTGCAt                                                                          <  1:511609‑M2/227‑1 (MQ=255)`  
                                                                                                                                                                                                                                          `aacgacactgacttctgacaggttgcttgaCAGTTGTTTCATATGAAGAGATGGTTAAGTCACGATAATCAATAGGAGAAATCAATAAGATCGTTTCTGACATCGAAGCTAACGCCCTCTTAGAGAGCGTCACTAGGTTCCACTGCGGGGTTATCTACGACTACTCCACCGCTGAGTACGTAAGCTACCGTCCGAGTGACTTCGGTGCGTATCTGGATGCGCTGGAAGCCGATGTTGCACGAGGCGGTCTTATTGTGTTCCACAACGGTCACAAGTATGACGTTCCTGCATTGACCAAACt                                                                <  1:165709‑M2/271‑1 (MQ=255)`  
                                                                                                                                                                                                                                                                `gcgactgaacgatgaggctcgtaaagaggccacacagtcacgATAATCAATAGGAGAAATCAATATGATCGTTTCTGACATCGAAGCTAACGCCCTCTTAGAGAGCGTCACTAAGTTCCACTGCGGGGTTATCTACGACTACTCCACCGCTGAGTACGTAAGTTACCGTCCGAGTGACTTCGGTGCGTATCTGGATGCGCTGGAAGCCGAGGTTGCACGAGGCGGTCTTATTGTGTTCCACAACGTTCACAAGTATGACGTTCCTGCATTGACCAAACTGGCAAATTTGCAATTGAACCga                                          >  1:752373‑M2/43‑301 (MQ=255)`  
                                                                                                                                                                                                                                                                `gcgactgaacgatgaggctcgtaaagaggccacacagtcacgATAATCAATAGGAGAAATCAATATGATCGTTTCTGACATCGAAGCTAACGCCCTCTTAGAGAGCGTCACTAAGTTCCACTGCGGGGTTATCTACGACTACTCCACCGCTGAGTACGTAAGCTACCGTCCGAGTGACTTCGGTGCGTATCTGGATGCGCTGGAAGCCGAGGTTGCACGAGGCGGTCTTATTGTGTTCCACAACGGTCACAAGTATgacgttcctgcattgaccaaactctgtctcttatacacatctccc                                          >  1:409391‑M2/43‑279 (MQ=255)`  
                                                                                                                                                                                                                                                                 `cgactgaacgatgaggctcgtaaagaggccacacagtcacgATAATCAATAGGAGAAATCAATATGATCGTTTCTGACATCGAAGCTAACGCCCTCTTAGAGAGCGTCACTAAGTTCCACTGCGGGGTTATCTACGACTACTCCACCGCTGAGTACGTAAGCTACCGTCCGAGTGACTTCGGTGCGTATCTGGATGCGCTGGAAGCCGAGGTTGCACGAGGCGGTCTTATTGTGTTCCACAACGGTCACAAGTATGACGTTCCTGCATTGACCCAACTGGCAAAGTTGCAATTGAACCgag                                         >  1:268580‑M2/42‑301 (MQ=255)`  
                                                                                                                                                                                                                                                                     `tgaCAGTTGTTTCATATGAAGAGATTGTTAAGTCACGATAATCAATAGGAGAAATCAATATGATCGTTTCTGACATCGAAGCTAACGCCCTCTTAGAGAGCGTCACTAAGTTCCACTGCGGGGTTATCTACGACTACTCCACCGCTGAGTACGTAAGCTACCGTCCGAGTGACTTCGGTGCGTATCTGGATGCGCTGGAAGCCGAGGTTGCACGAGGCGGTCTTATTGTGTTCAACAACGGTCACAAGTATGACGTTCCTGCATTGACTAAACTGGCAAAGTTGCAATTGAACCGAGAGtt                                     >  1:519005‑M2/4‑301 (MQ=255)`  
                                                                                                                                                                                                                                                                         `aGTTGTTTCATATGAAGAGATTGTTAAGTCACGATAATCAATAGGAGAAATCAATATGATCGTTTCTGACATCGAAGCTAACGCCCTCTTAGAGAGCGTCACTAAGTTCCACTGCGGGGTTATCTACGACTACTCAACCGCTGAGTACGTAAGCTACCGTCCGAGTGACTTAGGTGCGTATCTGGATGCGCTGGAAGCCGAGGTTGCACGAGGCGGTCTTATTGTGTTCAACAACGGTCACAAGTATGACGTTCCTGCATTGACCAAACTGGCAAAGTTGCAATTGAACCGAGAGTTCCAc                                 <  1:124370/301‑1 (MQ=255)`  
                                                                                                                                                                                                                                                                           `gtgggccggagatgtgtataagagacagacgATAATCAATAGGAGAAATCAATATGATCGTTTCTGACATCGAAGCTAACGCCCTCTTAGAGAGCGTCACTAAGTTCCACTGCGGGGTTATCTACGACTACTCCACCGCTGAGTACGTAAGCTACCGTCCGAGTGACTTCGGTGCGTATCTGGATGCGCTGGAAGCCGAGGTTGCACGAGGCGGTCTTATTGTGTTCCACAACGGTCACAAGTATGACGTTCCTGCATTGACCAAACTGGCAAAGTTGCAATTGAACCGAGAGTTCCACCt                               <  1:493737‑M2/270‑1 (MQ=255)`  
                                                                                                                                                                                                                                                                                       `aagaggccacacagtcacgATAATCAATAGGAGAAATCAATATGATCGTTTCTGACATCGAAGCTAACGCCCTC‑TAGAGAGCGTCACTAAGTTCCACTGCGGGGTTATCTACGACTACTCCACCGCTGAGTACGTAAGCTACCGTCCGAGTGACTTCGGTGCGTATCTGGATGCGCTGGAAGCCGAGGTTGCACGAGGCGGTCTTATTGTGTTCCACAACGGTCACAAGTATGACGTTCCTGCATTGACCAAACTGGCAAAGTTGCAATTGAACCGAGAGTTCCACCTTCCTCGTGAGAAc                  <  1:172994‑M2/282‑1 (MQ=255)`  
                                                                                                                                                                                                                                                                                        `agggaTTTTTAAGTAACGATAAGAAATAGGAGAAATCAATATGATCGTTTCTGACATCGAAGCTAACGCCCTCTTAGAGAGCGTCACTAAGTTCCACTGCGGGGTTATCTACGACTACTCCACCGCTGAGTACGTAAGCTACCGTCCGAGTGACTTCGGTGCGTATCTGGATTCGCTGGAAGCCGATGTTGCACTAGGCGGTCTTATTGTGTTCCACAACGGTCACAAGTATGACGTTCCTGCATTGACCAAACTGGCAAAGTTGCAATTGAACCGAGAGTTCCACCTTCCTCGTGAGAAc                  <  1:362802/298‑1 (MQ=255)`  
                                                                                                                                                                                                                                                                                         `gagaTTGTTAAGTCACGATAATCAATAGGAGAAATCAATATGATCGTTTCTGACATCGAAGCTAACGCCCTCTTAGAGAGCGTCACTAAGTTCCACTGCGGGGTTATCTACGACTACTCCACCGCTTAGTACGTAAGCTACCGTCCGAGTGACTTCGGTGCGTATCTGGATGCGCTGGAAGCCGAGGTTGCACGAGGCGGTCTTATTGTGTTCCACAACGGTCACAAGTATGACGTTCCTGCATTGACCAAACTGGCAAAGTTGCAATTGAACCGAGAGTTCCACCTTCCTCGTCAGAACt                 >  1:264887/1‑301 (MQ=255)`  
                                                                                                                                                                                                                                                                                           `ggccacacagtcacgATAATCAATAGGAGAAATCAATATGATCGTTTCTGACATCGAAGCTAACGCCCTCTTAGAGAGCGTCACTAAGTTCCACTGCGGGGTTATCTACGACTACTCCACCGCTGAGTACGTAAGCTACCGTCCGAGTGACTTCGGTGCGTATCTGGATGCGCTGGAAGCCGAGGTTGCACGAGGCGGTCTTATTGTGTTCCACAACGGTCACAAGTATGACGTTCCTGCATTGACCAAACTGGCAAAGTTGCAATTGAACCGAGAGTTCCACCTTCCTCGTGAGAACTGt               >  1:348852‑M2/16‑301 (MQ=255)`  
                                                                                                                                                                                                                                                                                            `aTTGTTAAGTCACGATAATCAATAGGAGAAATCAATATGATCGTTTCTGACATCGAAGCTAACGCCCTCTTAGATAGCGTCACTAAGTTCCACTGCGGGGTTATCTACGACTACTCCACCGCTGAGTACGTAAGCTACCGTCCGAGTGACTTCGGTGCGTATCTGGATGCGCTGGAAGCCGAGGTTGCACGAGGCGGTCTTATTGTGTTCCACAACTGTCACAAGTATGACGTTCCTGCATTGACCAAACTGGCAAAGTTGCAATTGAACCGAGAGTTCCACCTTCCTCGTGAGAACTGTa              >  1:79960/1‑301 (MQ=255)`  
                                                                                                                                                                                                                                                                                                   `agtaacgATAATCAATAGGAGAAATCAATATGATCGTTTCTGACATCGAAGCTAACGCCCTCTTAGAGAGCGTCACTAAGTTCCACTGCGGGGTTATCTACGACTACTCCACCGCTGAGTACGTAAGCTACCGTCCGAGTGACTTCGGTGCGTATCTGGATGCGCTGGAAGCCGAGGTTGCACGAGGCGGTCTTATTGTGTTCCACAACGGTCACAAGTATGACGTTCCTGCATTTACCAAACTGGCAAAGTTGCAGTTGAACCGAGAGTTCCACCTTCCTCGTGAGAACTGTATTGacac       <  1:807159‑M2/294‑1 (MQ=255)`  
                                                                                                                                                                                                                                                                                                    `gtcacgATAATCAATAGGAGAAATCAATATGATCGTTTCTGAAATCGAAGCTAACGCCCTCTTAGAGAGCGTCACTAAGTTCCACTGCGGGGTTATCTACGACTACTCCACCGCTGAGTACGTAAGCTACCGTCCGAGTGACTTCGGTGCGTATCTGGATGCGCTGGAAGCCGAGGTTGCACGAGGCGGTCTTATTGTGTTCCACAACGGTCACAAGTATGACGTTCCTGCATTGACCAAACTGGCAAAGTTGCAATTGAAACGAGAGTTCCACCTtcctcgtgagaacctgtcccttatt      >  1:432165‑M2/7‑289 (MQ=255)`  

                                                                                                                                                                                                                                                                                                          `|`                                                                                                                                                                                                                                                                                                            
`AGAACTTAGGCTGGACTTGGACAAGACAGACTGGCTGGAAGAAACCAGAAGTTACCAAGCTGAGTCATAAGGTGTTCGATAAGGACACTATGACCCACATCAAGGCTGGTGATTGGGTTAAGGTTGACATGGGAGTTGTTGGTGGATACGGCTACGTCCGCTCAGTTAGTGGCAAATATGCACAAGTGTCATACATCACAGGTGTTACTCCACGCGGTGCAATCGTTGCCGATAAGACCAACATGATTCACACAGGTTTCTTGACAGTTGTTTCATATGAAGAGATTGTTAAGTCACGATAATCAATAGGAGAAATCAATATGATCGTTTCTGACATCGAAGCTAACGCCCTCTTAGAGAGCGTCACTAAGTTCCACTGCGGGGTTATCTACGACTACTCCACCGCTGAGTACGTAAGCTACCGTCCGAGTGACTTCGGTGCGTATCTGGATGCGCTGGAAGCCGAGGTTGCACGAGGCGGTCTTATTGTGTTCCACAACGGTCACAAGTATGACGTTCCTGCATTGACCAAACTGGCAAAGTTGCAATTGAACCGAGAGTTCCACCTTCCTCGTGAGAACTGTATTGACACCCTTG  >  NC_001604.1/14033‑14629`

  

Alignment Legend

Aligned base mismatch/match (shaded by quality score): `ATCG/ATCG` `< 3 ≤` `ATCG/ATCG` `< 16 ≤` `ATCG/ATCG` `< 27 ≤` `ATCG/ATCG` `< 37 ≤` `ATCG/ATCG` `< 38 ≤` `ATCG/ATCG`

Unaligned base: `atcg`    Masked matching base: `atcg`    Alignment gap: `‑`    Deleted base: `‑`

Reads not counted as support for junction

read\_name Not counted due to insufficient overlap past the breakpoint.

read\_name Not counted due to not crossing MOB target site duplication.
